# No adaptation of a herbivore to a novel host but loss of adaptation to its native host

**DOI:** 10.1038/srep16211

**Published:** 2015-11-18

**Authors:** Amir H. Grosman, Adrián J. Molina-Rugama, Rondinelli Mendes-Dias, Maurice W. Sabelis, Steph B.J. Menken, Angelo Pallini, Johannes A.J. Breeuwer, Arne Janssen

**Affiliations:** 1 Institute for Biodiversity and Ecosystem Dynamics, University of Amsterdam, Science Park 904, 1098 XH Amsterdam, The Netherlands; 2 Department of Entomology, Federal University of Viçosa, Minas Gerais, Brazil

## Abstract

Most herbivorous arthropods are host specialists and the question is which mechanisms drive the evolution of such specialization. The theory of antagonistic pleiotropy suggests that there is a trade-off between adaptation of herbivores to a novel host and their native host. The mutation accumulation hypothesis proposes that herbivores on a novel host lose their adaptation to the native host through the accumulation of mutations with negligible effects on performance on the novel host. Experimental evidence for either of the two hypotheses is scarce. We compared the fitness of two sympatric moth strains from an introduced host and a native host. The strain from the novel host did not perform better on this host than the strain from the native host. The strain from the novel host performed less well on the native host than did the strain from the native host. Hence, selection on the novel host did not result in noticeable gain in performance, but adaptation to the native host was lost. These results are more readily explained by the mutation-accumulation hypothesis than by the trade-off hypothesis.

Two hypotheses predominate in the literature on the evolution of specialization in herbivorous arthropods, the first is based on the occurrence of trade-offs in performance on different hosts (antagonistic pleiotropy, a negative correlation in fitness over environments)^1^,^2^,^3^,^4^,^5^, and the second on mutation accumulation^6^,^7^. According to the trade-off hypothesis, different host plants impose different selection regimes on herbivores, which leads to different adaptations^2^,^3^,^8^,^9^,^10^, such that adaptations to one host results in a poorer performance on alternative hosts^1^,^3^,^11^ due to the antagonistic pleiotropy among genes^4^. These trade-offs are likely to involve many different life-history traits^2^, and are therefore not to be confused with trade-offs between life-history traits^12^,^13^. Trade-offs may explain the predominance of specialists among herbivorous insects^3^,^4^,^9^ and may play an important role in sympatric speciation due to disruptive selection^14^,^15^.

The hypothesis of mutation accumulation assumes that adaptation to a host and loss of adaptation to an alternative host are not causally related^7^. Instead, it hypothesizes that herbivores will become less adapted to alternative hosts when specializing on one host because of the stochastic accumulation of mutations that are neutral on the current host, but that result in reduced performance on the alternative hosts^6^,^7^. Because adaptation to the current host occurs simultaneously with the accumulation of mutations, but not necessarily at the same rate, this may result in a negative correlation between performance on the native host and the novel host, but the reduced performance on the native host not being the result of adaptation to the novel host. Thus, such negative correlation could erroneously be interpreted as evidence of a trade-off.

Both hypotheses ignore to some extent the (co-)evolutionary arms race between host plants and herbivores^16^, hence, the host plants themselves may also evolve in response to herbivory, resulting in reduced adaptation of the herbivores. When on a novel host plant, herbivores will participate in this arms race and will continue to adapt to this host. However, they will not adapt to the native host, which is evolving because of a coevolutionary arms race with other populations of herbivores. This process will also result in reduced adaptation of the herbivore to the native host, even without the accumulation of mutations in the herbivore and without a trade-off being involved. Hence, there are three processes that cause a herbivore becoming less adapted to alternative host plants: trade-offs in performance, mutation accumulation and evolutionary changes in the native host plant. The trade-off hypothesis predicts that (1) herbivores from the novel host plant will be better adapted to this host than the herbivores from the native host, whereas (2) the opposite is true for the herbivores from the native host plant. The latter two hypotheses always predict point (2), but not necessarily point (1). Hence, the latter two processes will be difficult to distinguish in practice.

Despite extensive research, there is not much evidence for mutation accumulation^17^,^18^ or for trade-offs^3^,^4^,^9^ (but see Cooper and Lenski^19^ and Magalhães *et al.*^20^,^21^). As concerns trade-offs, this may partly be caused by the experimental methods used^4^. For example, Kawecki and Ebert^22^ point out that local adaptation should be verified by showing that each local population is superior in its own habitat to populations from other habitats. Thus, the performance of several populations need to be tested in their own habitats as well as in the foreign habitats (the so-called “local vs. foreign” criterion)^22^. In many cases, trade-offs were studied, however, by comparing the performance of herbivores from one host plant on several other host plant species^10^,^23^,^24^,^25^,^26^,^27^,^28^,^29^. Often, herbivores perform relatively well on non-hosts, which is considered as evidence for the lack of trade-offs. However, when herbivores are artificially placed on a novel host for one or a few generations, adaptation to the new host may not have occurred yet, and trade-offs with old adaptations are therefore still not expressed. Moreover, good performance on a novel host plant is not necessarily the result of adaptation; such a pattern may also result from similarity between the native and the novel host plant^30^,^31^. Performance may be even higher on a novel host plant due to its superior quality, for example because of higher nutrient availability^22^. Adaptation has only occurred when performance of the herbivore on the novel host plant has improved over generations: when herbivores that switched to a novel host perform better on that host than conspecifics that remained on the native host. Trade-offs, likewise, can only have occurred when a population that adapted to a novel host consequently performs less well on the native host—to which it was previously adapted—than the strain from the native host. This furthermore only holds if the strain on the native host did not adapt further to it since the moment that the other strain moved to the novel host. Even then, the loss of adaptation to the native host is not necessarily caused by the adaptation to the novel host, but could simply arise from accumulation of mutations with negative effects on the performance on the native host or with evolutionary changes in the native host.

Natural systems that are suitable for studying adaptation to host-plants are those where herbivores naturally occur on several hosts for a period that has been sufficient for adaptation to take place^11^,^32^,^33^,^34^. In systems in which the alternative host plants occur in sympatry^11^,^32^,^34^, gene flow between populations is a potential obstacle for novel adaptation^14^,^15^,^22^. Hence, such systems are especially valuable for testing the scenario of sympatric speciation^14^,^15^. Here, we investigate the adaptation of a herbivore to a novel host plant and the performance on its native host plant in order to detect signs of trade-offs, mutation accumulation in the herbivore or evolution of the native host plant.

We studied a large scale semi-natural experiment which is ideally suited to test if (loss of) adaptation and trade-offs can take place in the face of gene flow within a relatively short time period. About 10 species of *Eucalyptus* were introduced from Australia into southern Brazil around 1905^35^ and eucalyptus forests now cover about 7 million hectares. Eucalyptus foliage is renowned for its variety of secondary metabolites^36^,^37^,^38^, and it was therefore expected that eucalyptus would be well protected from herbivores. However, many insect herbivores (mainly Lepidoptera, but also Coleoptera and leaf-cutter ants) that used native Myrtaceae such as guava (*Psidium guajava* L.) and other native host plants, readily colonized eucalyptus^39^,^40^,^41^,^42^. Currently, *Thyrinteina leucoceraea* Ringe (Lepidoptera: Geometridae) is considered one of the primary pests in eucalyptus plantations^40^, including *Eucalyptus grandis* Hill ex. Maiden (Myrtaceae). Yet, it is not a pest on guava, its native host plant, which occurs in sympatry with eucalyptus.

We measured performance of the herbivore on *E. grandis* and on guava. To do so, we estimated the performance of *T. leucoceraea* from sympatric populations from each of the two host plants on material of both plant species from the same locations as the herbivores. Because studies considering both larval and adult performance parameters have a higher likelihood of detecting trade-offs^4^,^43^, we measured several life history traits (developmental period and survival of larvae and pupae, pupal weight and fecundity). We then combined most of these traits in two proxies for fitness, the net reproductive rate (*R*_*0*_) and the intrinsic rate of increase (*r*_*m*_)^32^,^44^,^45^. We found that both strains performed better on the native host than on the novel host, and the strain from the novel host did not perform better on the novel host than did the strain from the native host. Moreover, the strain from the novel host performed less well on the native host than did the strain from the native host.

## Results

**Figure 1:**
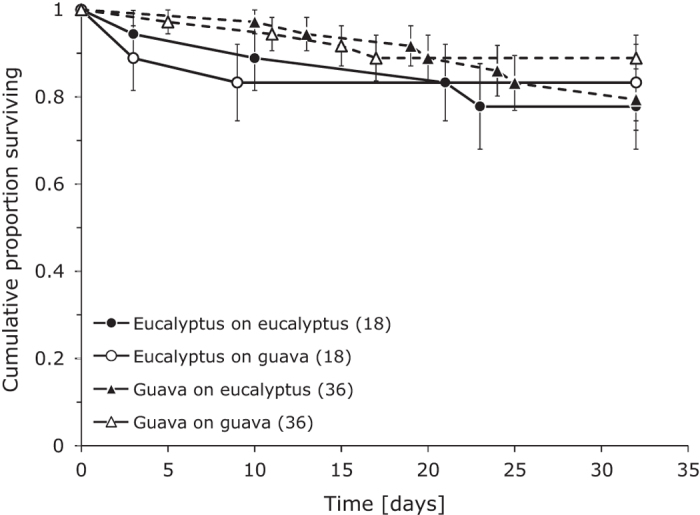
Kaplan Meier survivorship of herbivores of the guava line (dashed lines) and the eucalyptus line (drawn lines) on guava (white symbols) and on eucalyptus (black symbols). Data were pooled over sexes. Numbers between brackets are numbers of replicates. There were no significant differences in survival among treatments.

Survival until adulthood was generally high (Fig. 1), and there was no significant difference in immature survival between the two strains (Table 1). Survival did not differ significantly on the two host plants and the effect of the interaction between strain and host plant on survival was also not significant (Fig. 1, Table 1). It should be noted that a trade-off between performance on the novel host and that on the native host results in an interaction between strain and host plant, but a significant interaction between these factors does not necessarily indicate the occurrence of a trade-off^22^.

**Figure 2:**
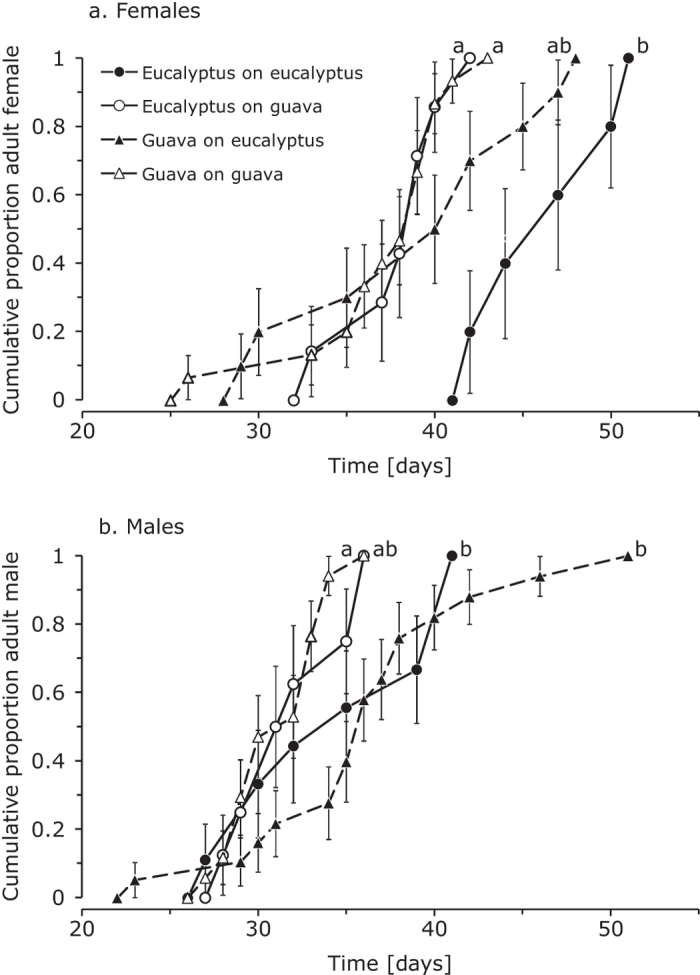
Cumulative proportion of herbivores originating from guava (dashed line) and eucalyptus (drawn line) reaching adulthood when feeding on guava (black symbols) or on eucalyptus (white symbols). Development of females (**a**) and males (**b**) is presented separately. Per panel, lines with different letters are significantly different.

Females reached adulthood significantly later than males (Fig. 2, Table 1). Females of the guava strain developed significantly faster than females from the eucalyptus strain (Table 1), the difference in developmental rate on the two host plants was marginally significant, as was the effect of the interaction between host plant and strain (Table 1). Females of the eucalyptus strain developed significantly slower on eucalyptus than on guava (Fig. 2a), females of the guava strain also developed slower on eucalyptus than on guava, but this trend was not significant. Females of both strains developed equally fast on guava (Fig. 2a).

The developmental rate of males of the two strains did not differ significantly (Table 1), development on eucalyptus was slower than on guava (Fig. 2b), but this difference was only marginally significant (Table 1). The interaction between strain and host plant had no significant effect on male developmental rate (Table 1). Males of the guava strain developed significantly slower on eucalyptus than on guava (Fig. 2b). The same trend, though not significant, was observed for males of the eucalyptus strain. Males from both strains developed equally fast on guava (Fig. 2b).

**Figure 3:**
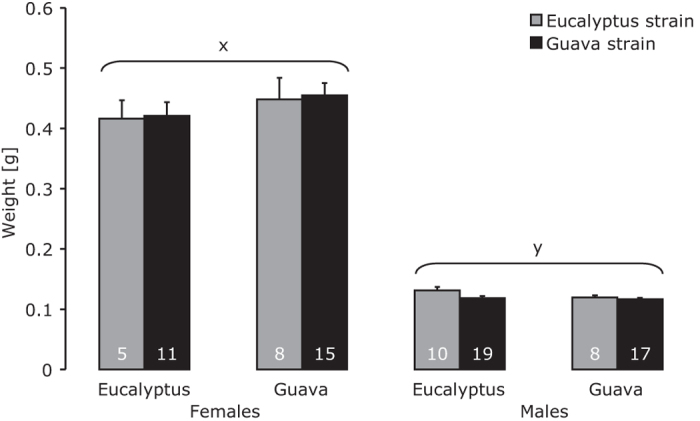
Average pupal weight (±SE) of male and female *T. leucoceraea* from guava (black bars) and eucalyptus (grey bars) fed with guava or eucalyptus. Numbers inside bars are number of replicates. Bars marked with different letters differ significantly.

The pupal weight of females was significantly higher than that of males (Fig. 3, Table 1). The weight of females or males did not vary significantly with strain or with host plant, nor with the interaction between these two factors (Fig. 3, Table 1).

**Figure 4:**
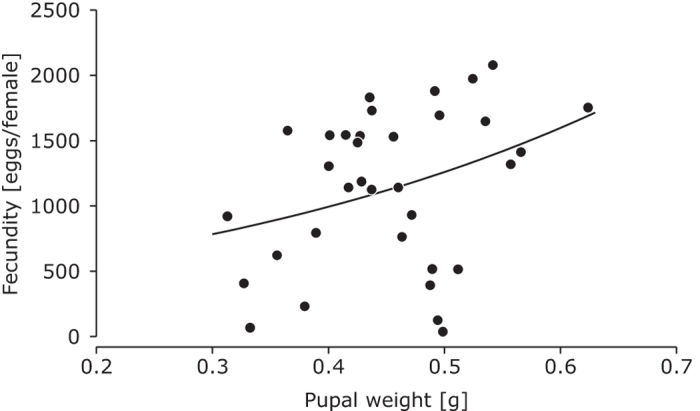
Relationship between weight of female pupae and the total number of eggs produced by females when adults. The curve is fecundity = exp(a + b*weight); a = 5.95 (s.e. = 0.56), b = 2.37 (s.e. = 1.20) (GLM with a quasi-Poisson error distribution).

There was no significant effect of host plant or strain on female fecundity, nor was there a significant interaction between these factors (Table 1). The average fecundity was higher for insects that consumed guava (guava = 1236 ± 142, n = 21, eucalyptus = 915 ± 127, n = 15), but this difference was not significant (Table 1). The fecundity of females increased almost significantly with their pupal weight (Fig. 4, GLM, *F*_1,32_ = 3.90, *P* = 0.057), but weight explained only 8.8% of the variation in fecundity.

**Figure 5:**
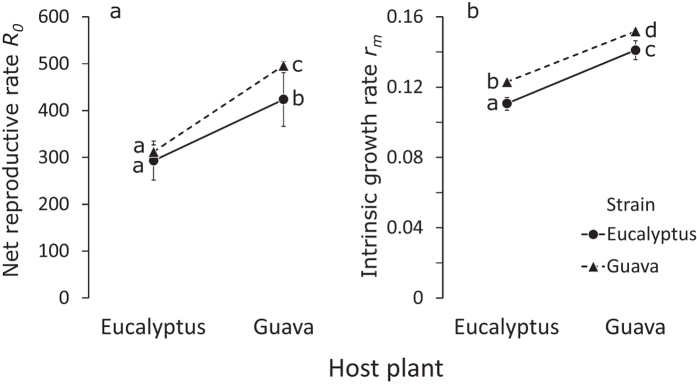
Average (±95% confidence intervals after jackknifing) net reproductive rate (*R*_*0*_, **a**) and intrinsic growth rate (*r*_*m*_, **b**) of the eucalyptus and guava strain on a diet of eucalyptus and guava host plant foliage. Different letters next to the data indicate significant differences based on the lack of overlap of the confidence intervals, confirmed with a GLM on the jackknife values. Including egg fertility into the data yielded qualitatively similar results (data not shown).

There was a significant effect of the interaction between herbivore strain and host plant on the net reproductive rate (Table 2, Fig. 5a), but this interaction was not caused by a trade-off in performance. Rather, both strains performed equally well on eucalyptus, the novel host (Fig. 5a), but the eucalyptus strain performed less well on a diet consisting of guava foliage than did the guava strain (Fig. 5a). Both strains performed better on guava than on eucalyptus (Table 2, Fig. 5a).

There was no significant effect of the interaction between strain and host plant on the intrinsic growth rates (Table 2, Fig. 5b). The guava strain performed significantly better on eucalyptus and on guava than did the eucalyptus strain (Fig. 5b, Table 2), and both strains performed better on guava than on eucalyptus (Fig. 5b, Table 2).

## Discussion

We evaluated juvenile survival, developmental rate, pupal weight and fecundity to find evidence for adaptation of a strain of the phytophagous moth *T. leucoceraea* to its novel host plant, eucalyptus. Adaptation would be manifest when this strain collected from the novel host would perform better on this host than a strain collected from the native host. Neither of the parameters separately provided evidence for better adaptation of the eucalyptus to the novel host plant than the guava strain.

When most of these parameters were combined to calculate the net reproductive rate and the intrinsic growth rate, two much-used fitness estimates^44^,^45^, we also did not find significant trends for the eucalyptus strain being more adapted to eucalyptus than the guava strain (Fig. 5). Rather, on eucalyptus, the intrinsic growth rate of the guava strain was higher than that of the eucalyptus strain (Fig. 5b). No such significant difference was found for the net reproductive rate (Fig. 5a). The fitness estimates of the guava strain on guava were significantly higher than those of the eucalyptus strain on guava (Fig. 5).

The two proxies for fitness concern the fitness of female offspring, not that of males. Perhaps males from the eucalyptus strain perform better than males from the guava strain, thus compensating for fitness differences in the females. However, we found no significant differences in male life-history parameters between the two strains (Table 1).

A much-used other measure of herbivore performance is size or weight of the pupae or adults^46^,^47^,^48^. We found a positive relationship between female pupal weight and fecundity but the regression was not significant, and weight explained only a small proportion of the variation in fecundity (Fig. 4). We furthermore found weak correlations of pupal weight with individual net reproductive rates and intrinsic growth rates (see Supplementary Information for calculations and results). This is further evidence that size-fitness relationships are not sufficiently reliable to consider size as a measure for herbivore performance^22^,^46^,^47^,^48^.

The reason for the lack of adaptation of the herbivore to the novel host remains unclear. Possibly, the time since *T. leucoceraea* has colonized eucalyptus was insufficient to adapt to the novel host plant. The first report of *T. leucoceraea* in eucalyptus plantations is from 1977^49^, 28 years before this study was carried out. According to our data, *T. leucoceraea* has a generation time of 41–51 days at 25 °C, *i.e.* c. 7–9 generations per year. The mean annual temperature in our study area (Viçosa, Minas Gerais, Brazil) is 20.7 °C^50^, thus generation time will be somewhat longer in the field than in our lab experiment. Taking 6 generations of *T. leucoceraea* per year under field conditions as a reasonable estimate, this herbivore has been on eucalyptus for at least 168 generations. It is likely that *T. leucoceraea* had colonized eucalyptus long before 1977, when it was first reported from eucalyptus plantations. Hence, *T. leucoceraea* has most likely been on eucalyptus for several hundred generations. In the apple maggot fly *Rhagoletis pomonella*, such a number of generations was sufficient for adaptation to a novel host plant and host race formation, and partial reproductive isolation has occurred between populations from native and novel host plants in this species^34^. Thus in theory, adaptation of *T. leucoceraea* to eucalyptus could have occurred since the introduction of this host plant in Brazil.

Both fitness measures show that the eucalyptus strain performed significantly worse on the native host plant than did the guava strain. Hence, rather than having become less adapted to the native host as a consequence of adaptation to the novel host (trade-off or antagonistic pleiotropy), the loss of adaptation to the native host seems to have preceded the adaptation to the novel host (Fig. 5). This is more readily explained by mutation accumulation hypothesis^6^,^7^,^17^,^18^,^19^ or by evolutionary changes in the native host plant. Because of the difference in generation time of the native host tree and the herbivore, we suspect that mutation accumulation in the herbivore is the most likely explanation. In this case, there is no trade-off between performance on the novel and native host, but the eucalyptus strain has lost its adaptation to the native host through the accumulation of mutations in genes that are no longer under pressure of selection imposed by the native host plant^7^,^19^. Such loss of adaptation renders the herbivores from the novel host plant less competitive on the native host plant, and this mechanism alone may result in host plant specialization^7^. Alternatively, it is possible that the eucalyptus strain made the very transition to the novel host plant because it consisted of individuals that performed less well on the native host and moved to the novel host to avoid competition with better-performing conspecifics^14^. However, densities of caterpillars of both populations are extremely low in the study area: it often took several hours of searching on host plants to find any caterpillar at all (Grosman and Janssen, pers. obs.). It therefore seems unlikely that intraspecific competition will have played a role in host plant selection.

If mutation-accumulation is responsible for the lower performance of the eucalyptus strain on guava, the adaptation to the novel host plant is apparently a slower process than the loss of adaptation to the native host due to the absence of selection against mutations in traits that are vital on the native host. This may function as an ecological trap, restricting the possibilities of herbivores on novel hosts to return to the native host where they may have to compete with better adapted conspecifics.

Another reason for the failure to detect trade-offs in performance are allocation constraints^51^: individuals of the guava strain may have more resources to allocate than individuals from the eucalyptus strain. However, we measured performance of both strains of the herbivores from egg to adult on the two different host plants, hence, such higher availability of resources could then only be realized through higher quality of the eggs with which the experiments were started. We did not find evidence for higher performance of the juveniles of the guava strain than those of the eucalyptus strain (Fig. 1), so we consider this explanation unlikely.

The remaining question is why the eucalyptus strain of *T. leucoceraea* did not remain on guava, which is the better host. There may also be other advantages associated with eucalyptus, for example, lower risks of predation or parasitism (i.e. enemy free space)^52^, or improved immune functions against pathogens or parasitoids^53^. These alternatives will be the focus of future work.

## Materials & Methods

### Cultures

Plant material for the *T. leucoceraea* cultures was obtained by growing seedlings, obtained from commercial growers, outside the laboratory until they were 3–6 months old. *Thyrinteina leucoceraea* densities in the field were low (Grosman and Janssen, pers. obs.) and collections did not yield sufficient individuals to perform experiments with field-collected individuals. Therefore, cultures were established by collecting caterpillars from two areas with guava and two with eucalyptus (*E. grandis*) on the campus of the Federal University of Viçosa, Minas Gerais, Brazil (20°45′ S, 42°51′ W). The distance among the areas was 0.5–2 km. The laboratory cultures were regularly supplemented with individuals from the field. Caterpillars from the two plant species were treated as separate strains (coined the guava and eucalyptus strain). Hence, each strain consisted of caterpillars from two areas with the same plant species, and was reared on the original host plant species. Caterpillars were reared either in groups within cages or individually in plastic cups. The rearing cages (70 × 70 × 100 cm) were placed outside the laboratory, and contained intact host plant seedlings. The rearing cups (plastic 500-ml cups closed with fine gauze) were kept in the laboratory at ambient temperature (c. 25 °C) and were exposed to natural light conditions (13L:11D). The cups contained small twigs of the host plant, of which the stems were inserted into moist vermiculite on the bottom of the cups to maintain leaf turgor. New twigs were added twice per week. Pupae were transferred to cages containing host plants and a small piece of moist filter paper dipped in a solution of honey in water (10% w/v). In these cages, adults emerged, mated and the females oviposited. Eggs were collected from the cages once a week, and were left to hatch in a rearing cage (see above).

### Survival, development and pupal weight

Per herbivore strain, six pairs of male and female pupae were randomly selected from the cultures. Owing to rearing limitations, three of the six pairs from the eucalyptus strain were selected two weeks after the other pairs. However, almost none of the larvae of these later pairs survived to adulthood, contrary to the offspring of the first three pairs. Therefore, we omitted the data of the larvae of this later cohort from the analysis of survival, development, fecundity and pupal weight.

Each pair was allowed to emerge, mate and oviposit in a cylindrical cardboard cage (50 cm height/20 cm diameter). The cages were positioned inside the laboratory at ambient temperature (25 °C) and were exposed to natural light conditions (13L:11D). Inside the cage, we placed a fresh leaf of the same plant species as consumed by the herbivores during the larval period. A piece of cotton wool, moistened with a solution of honey in water (10% w/v), was placed on the bottom of the cage. Eggs were collected from the cage and incubated (25 °C 13L:11D) until emergence in a plastic cup (500 ml) closed with fine gauze. Twelve first-instar caterpillars were collected 1–12 hours after emergence from the batch produced by each adult pair. The caterpillars were placed individually in Petri dishes (14 cm diameter) containing a eucalyptus or guava leaf (six caterpillars per diet). We used leaves from six eucalyptus and six guava trees from the areas mentioned above (see ‘cultures’). From the third instar on, caterpillars were reared individually in plastic cups containing a leaf, as used in the cultures (see above). We scored survival of the caterpillars daily and replaced leaves that had visibly lost turgor. Each caterpillar consumed leaves of the same tree during the entire experiment. Pupae were weighed (Precisa 262SMA-FR, Precisa Instruments Ltd, Switzerland) and their sex was determined one day after pupation.

The cumulative proportion of herbivores surviving until adulthood was analysed with a time-to-event analysis (Cox proportional hazards model of the “coxme” package of R 2.12^54^) with host species and strain as fixed factors and parent pair and tree individual from which the leaves were collected as random factors. Developmental rates from 1^st^ instar larva to adulthood were analysed with a similar model with the same factors, except that gender was included as extra fixed factor, because developmental rates of males and females often differ in arthropods. We used a linear mixed effects model (lme of the package nlme^55^) for the analysis of pupal weight (log-transformed), with gender, host plant and strain as fixed factors and parent pair and tree individual as random factors. In all analyses, we started with a full model including interactions of all fixed factors, and subsequently removed non-significant interactions and factors until a minimal adequate model was reached.

### Fecundity

The pupae obtained in the above experiment were incubated in a climate box until emergence (±25 °C; 12L: 12D). Approximately twelve hours after emergence, male and female adults were paired within each treatment. The pairs were allowed to mate and the females oviposited under the same conditions as in the survival and development experiment (see above). After the females had died, the eggs were collected from the cage and counted.

Differences in adult fecundity were tested with a linear mixed effects model with host plant and strain as fixed factors and parent pair and tree individual as random factors. The relation between pupal weight and fecundity was initially tested with a similar model with pupal weigh, host plant and strain as fixed factors and parent pair and tree individual as random factors. Host plant, strain, parent pair and tree proved to be not significant, and we therefore used a GLM with a quasi-Poisson error distribution with pupal weight as factor.

### Net reproductive rate and intrinsic growth rate

The net reproductive rate is the product of the age-specific survival rate and oviposition rate corrected for the offspring sex ratio (proportion females). The intrinsic growth rate uses this same product but also incorporates the developmental rate and the timing of the oviposition events^56^,^57^. We estimated the net reproductive rate (*R*_*0*_) and the intrinsic growth rate (*r*_*m*_) using the survival rate of the entire cohort of each treatment (Fig. 1), and the developmental rate (Fig. 2), fecundity data and offspring sex ratio of the individual females (above), further assuming that the pre-oviposition period was 3 days and the oviposition period was 6 days (based on A.H. Grosman, pers. obs.) for all treatments. We used the Lotka-Euler equation to estimate the intrinsic growth rate^56^,^57^. A jackknife method was used to obtain means and confidence intervals of the two rates for each treatment. This method involves the calculation of both rates for the entire population minus one individual, and this process is repeated with each individual being left out in turn. Averages were considered significantly different when the 95% confidence intervals did not overlap. We further verified this with a GLM on the jackknife values with host plant and strain as factors, a Gaussian error distribution for the intrinsic growth rate and a quasi-Gaussian distribution for the reproductive rate.

## Additional Information

**How to cite this article**: Grosman, A. H. *et al.* No adaptation of a herbivore to a novel host but loss of adaptation to its native host. *Sci. Rep.* **5**, 16211; doi: 10.1038/srep16211 (2015).

## Supplementary Material

Supplementary Information

## Figures and Tables

**Table 1:** Results of statistical tests of life-history parameters of the guava and eucalyptus strains of the geometrid moth *Thyrinteina leucoceraea* on eucalyptus and guava hosts.

Parameter	Gender		Strain x Host		Strain		Host	
	value	*P*	value	*P*	value	*P*	value	*P*
Survival^a^	n.a.		0.056	0.81	0.84	0.36	0.25	0.61
Development^a^	55.5	**<0.001**						
Female	n.a.		3.64	0.057	4.17	**0.041**	3.00	0.083
Male	n.a.		1.13	0.29	0.88	0.35	3.82	0.051
Pupal weight^b^	282.2	**<0.001**						
Female			0.74	0.39	0.081	0.78	2.24	0.13
Male			1.98	0.16	2.84	0.092	1.84	0.18
Fecundity^b^	n.a.		0.39	0.53	0.74	0.39	2.63	0.11

Whenever there was a significant effect of gender on the parameter, separate analyses were done for males and females. Values of *P* < 0.05 are given in bold. Gender was not included in the analysis of survival until adulthood because it could not be assessed for individuals that died before turning adult. All degrees of freedom are 1.

^a^Cox proportional hazards mixed effects, value is Chi^2^.

^b^Linear mixed effects model, value is log likelihood ratio. N.a.: not applicable.

**Table 2:** Results of statistical tests of performance of the guava and eucalyptus strains of the geometrid moth *Thyrinteina leucoceraea* on eucalyptus and guava hosts.

Parameter	Strain × Host		Strain		Host	
	*F*_*1,31*_	*P*	*F*_*1,33*_	*P*	*F*_*1,32*_	*P*
R_0_	6.16	**0.019**	26.7	**<0.001**	278.9	**<0.001**
r_m_	0.49	0.49	122.1	**<0.001**	746.7	**<0.001**

Results are from a generalized linear model with a quasi-Poisson error distribution. Values of *P *< 0.05 are given in bold.
