# Supplementary material for: No adaptation of a herbivore to a novel host but loss of adaptation to its native host
Source: Sci Rep. 2015 Nov 18;5:16211. doi: 10.1038/srep16211 (PMC4649677; doi:10.1038/srep16211)
Supplement: Supplementary Information [file srep16211-s1.doc]

**Supplementary information**

No adaptation of a herbivore to a novel host but loss of adaptation to its native host

Amir H. Grosman^1a^, Adrián J. Molina-Rugama^2^, Rondinelli Mendes-Dias^2^, Maurice W. Sabelis^1†^, Steph B.J. Menken^1^, Angelo Pallini^2^, Johannes A.J. Breeuwer^1^, Arne Janssen^1*^

**Pupal weight is not a good proxy for fitness**

Pupal weight, adult weight and adult size are often used as stand-in measures for fitness ^1–3^. Because we measured pupal weight and also estimated net reproductive rate and intrinsic growth rate, we could verify whether pupal weight indeed correlated with these latter two measures of fitness. However, both reproductive rate and intrinsic growth rate cannot be calculated for individuals, but only for cohorts. As a way around this, we estimated the relative (reproductive and intrinsic) rate for each individual *i* as the difference between the rate for the entire group (*R_0_* and *r_m_*) and the rate for the group without this individual (i.e. *R_0-i_* and *r_m-i_*). The difference between the population estimate of the rates (*R_0_* and *r_m_*) and the estimate of the population without an individual *i* (*R_0-i_* and *r_m-i_*) is thus an estimate of the relative contribution of individual *i* (hence *R_0_* – *R_0-i_* and *r_m_* – *r_m-i_*), with positive values indicating an above-average contribution of individual *i* to the population average. We calculated these relative rates using the *r_m_* of the entire data set (hence, each of the two herbivore populations on both host plants) and regressed them with pupal weight to investigate whether the weight of an individual is a good predictor for relative fitness.

The regression between pupal weight and the relative net reproductive rate (*R_0_* – *R_0-i_*) was bordering significance (*F*_1,32_ = 4.02, *P* = 0.054) but pupal weight accounted for only 8.4 % (adjusted R^2^) of the variation (Supplementary Figure 1a). The regression between pupal weight and *r_m_* – *r_m-i_* was significant (*F*_1,32_ = 4.87, *P* = 0.035) but pupal weight accounted for only 10.5 % of the variation (Supplementary Figure 1b). This is further evidence that size-fitness relationships are not sufficiently reliable to consider size as a measure for herbivore performance ^1–3^.

**Supplementary Figure S1.** The relationship between pupal weight of individual female moths and the relative net reproductive rate (*R_0_* – *R_0-i_*, a) and the relative intrinsic rate of increase (*r_m_* – *r_m-i_*, b) of these same individuals. Lines are linear regression lines, the equations and the adjusted *R^2^* are given in the figures. Leaving out the obvious outlier in (b) resulted in a stronger regression (F_1,31_ = 7.29, *P* = 0.011, adjusted *R^2^* = 0.164). See text for further explanations.

**Genetic identity**

Genetic identity of the two cultures was determined by sequencing a portion of the mitochondrial CO1 gene. Separate DNA was isolated from 8 and 5 individuals of the two guava areas and 6 and 1 individuals from the eucalyptus areas using the chelex method. DNA was isolated in 5% chelex and 5 μL proteinase K (20mg/μL). Zirconium beads were added and tissue was macerated. The tubes were then incubated at 65°C for 30 min followed by incubation at 96 for 10 min. A 700 bp portion of the mtCOI was amplified in a 20 µL reaction volume consisting of 8.2 µL H_2_O, 4.0 µL 5 X PCR buffer, 4.0 µL 1 mM dNTP’s, 0.6 µL 10 mg/ml bovine serum albumin, 0.4 µL 10 µM primers (forward and reverse), 0.4 µL 5U/µL “Phyre Hot Start II” polymerase (Finnzymes, Finland) and 2.0 µL template DNA. PCR cycling conditions were: an initial denaturation step at 98°C for 10 s was followed by an annealing step at 55°C for 30 sec. A cycle of denaturation at 98°C for 10 s, annealing at 55°C for 10 s and elongation at 72°C for 20 s was repeated 35 times. The PCR was concluded by an elongation step at 72°C for 5 min and a cooling step at 4°C for 5 min. Finally, 3 µL of PCR products was loaded on a 1 % agarose gel in order to control whether primer dimers had formed and whether the amplified gene fragments were of the required size. PCR products were sent for sequencing to MacroGen (Amsterdam).

A BLASTn search in GenBank was executed to confirm that sequences were indeed COI fragments of Lepidoptera. Furthermore, sequences were translated into amino acids to ensure that stop codons were absent in the amplified DNA fragments using an invertebrate mitochondrial DNA translation table. Sequences were analysed and modified in CodonCode Aligner (CodonCode Inc.). Reviewing the chromatogram data in the Trace window, the 5’ and 3’ 20-200 bases of each sequence were deleted because of poor sequencing quality. Forward and reverse sequences were assembled in contigs. Ambiguous positions in the sequences – possibly a display of heterozygosity at these bases – were marked with nucleotide ambiguity codes (IUPAC). High quality sequences were imported in MEGA 5.2.1, where they were aligned using ClustalW and trimmed to equal lengths ^4^. Using the Neighbor-Joining method and p-distances, trees were constructed using MEGA software, applying 500 bootstrap replications to test the robustness of the phylogeny reconstruction. Codon positions included were 1st+2nd+3rd+Noncoding. All positions with less than 95% site coverage were eliminated. That is, fewer than 5% alignment gaps, missing data, and ambiguous bases were allowed at any position. Gaps and missing data were completely removed from the analysis. In order to root the tree, the first hits when BLASTing the sequences were downloaded from GenBank.

A taxonomic search in Genbank for *T. leucoceraea* showed that no information on this species was available. A BLASTn search of *T. leucoceraea* sequences in GenBank in June 2013 returned sequences from the sister species *T. arnobia* (92% maximum identity), which confirmed that our specimens belonged to the expected moth genus *Thyrinteina*. The outgroup consisted of the closest BLAST hits other than *Thyrinteina* accessions were used: *Holochroa* sp. *ochra* and *Eusarca* *nemora*. All accessions of this outgroup belonged to the same subfamily Ennominae in the family Geometridae to which the genus *Thyrinteina* belongs. The final dataset involved 30 nucleotide sequences and there were a total of 563 positions. Phylogenetic analysis suggested that this moth genus can be divided into three monophyletic clades (Figure S2). One clade contained all the specimens of this study that were morphologically identified as *T.*  *leucoceraea*. A second clade represented *T. arnobia* including all *T. arnobia* accessions from genbank. A third clade contained two specimens collected from French Guiana that were identified to the genus level, but species status has not been determined. Sequence variation for COI within the *T. leucoceraea* is almost absent (p-distance = 0.0004) and does not allow for a distinction between *T. leucoceraea* cultures based on host plant species, if host races existed at all.

**Figure S2.** A phylogenetic tree was inferred using the Neighbor-Joining method based p-distance. The tree is rooted with outgroup species *Holochroa* *ochra* and *Eusarca nemora*. The percentage of replicate trees in which the associated taxa clustered together in the bootstrap test (500 replicates) are shown next to the branches, only bootstrap values higher than 80% are shown. The tree is drawn to scale, with branch lengths of the number of base differences per site.

**References**

1. Leather, S. R. Size, reproductive potential and fecundity in insects - things aren’t as simple as they seem. *Oikos* **51,** 386–389 (1988).

2. Awmack, C. S. & Leather, S. R. Host plant quality and fecundity in herbivorous insects. *Annu. Rev. Entomol.* **47,** 817–844 (2002).

3. Moreau, J., Benrey, B. & Thiery, D. Assessing larval food quality for phytophagous insects: are the facts as simple as they appear? *Funct. Ecol.* **20,** 592–600 (2006).

4. Tamura, K. *et al.* MEGA5: Molecular evolutionary genetics analysis using maximum likelihood, evolutionary distance, and maximum parsimony methods. *Mol. Biol. Evol.* **28,** (2011).
